# Development and delivery of patient treatment in the Trondheim Hip Fracture Trial. A new geriatric in-hospital pathway for elderly patients with hip fracture

**DOI:** 10.1186/1756-0500-5-355

**Published:** 2012-07-16

**Authors:** Ingvild Saltvedt, Anders Prestmo, Elin Einarsen, Lars Gunnar Johnsen, Jorunn L Helbostad, Olav Sletvold

**Affiliations:** 1 Department of Geriatrics, St. Olav Hospital, University Hospital of Trondheim, Trondheim, Norway; 2 Departments of Neuroscience, Norwegian University of Science and Technology (NTNU), Trondheim, Norway; 3 Department of Orthopaedics, St. Olav Hospital, University Hospital of Trondheim, Trondheim, Norway

**Keywords:** Hip fractures, Geriatric assessment, Oldest old, Randomized controlled trial, Interdisciplinary health team

## Abstract

**Background:**

Hip fractures are common among frail elderly persons and often have serious consequences on function, mobility and mortality. Traditional treatment of these patients is performed in orthopedic departments without additional geriatric assessment. However, studies have shown that interdisciplinary geriatric treatment may be beneficial compared to traditional treatment. The aim of the present study is to investigate whether treatment of these patients in a Department of Geriatrics (DG) during the entire hospital stay gives additional benefits as compared to conventional treatment in a Department of Orthopaedic Surgery (DOS).

**Findings:**

A new clinical pathway for in-hospital treatment of hip fracture patients was developed. In this pathway patients were treated pre-and postoperatively in DG. Comprehensive geriatric assessment was performed as an interdisciplinary, multidimensional, systematic assessment of all patients focusing on each patient’s capabilities and limitations as recommended in guidelines and systematic reviews. Identification and treatment of co-morbidities, pain relief, hydration, oxygenation, nutrition, elimination, prevention and management of delirium, assessment of falls and osteoporosis were emphasized. Discharge planning started as early as possible. Initiation of rehabilitation with focus on early mobilisation and development of individual plans was initiated in hospital and continued after discharge from hospital. Fracture specific treatment was based upon standard treatment for the hospital, expert opinions and a review of the literature.

**Conclusion:**

A new treatment program for old hip fracture patients was developed, introduced and run in the DG, the potential benefits of which being compared with traditional care of hip fracture patients in the DOS in a randomised clinical trial.

## Findings

### Background

Hip fractures are common in elderly people [1]. Patients with hip fractures are heterogeneous with respect to age, pre-fracture function and morbidity [2,3]. Many are frail, have chronic comorbid disorders, cognitive impairment, low body weight and are functionally impaired before the fracture [3]. On admission to hospital they frequently suffer from concurrent minor or major medical conditions that may impact on prognosis [4,5].

After hip fractures a high proportion of the patients experiences reduced performance of basic and instrumental activities of daily living, reduced mobility with need of walking aids, decreased ability to move outside their own home, and they often report deterioration of health status [6-8]. A considerable proportion needs nursing home placement [7,8] and one-year mortality is high [1].

The Research Group on Geriatrics, St Olav Hospital, University Hospital of Trondheim, Norway, has previously performed a randomised clinical trial showing that by treating acutely sick, frail elderly patients in a geriatric evaluation and management unit, mortality was significantly reduced and the chance of living at home was improved [9-11]. Over years the group has also been focusing on research on assessment and treatment of older persons at risk of falling [12].

In 2007 the research group decided to perform a prospective randomised trial on treatment of hip fracture patients in order to investigate if treatment in the Department of Geriatrics (DG) can improve outcomes as compared to standard treatment in the Department of Orthopaedic Surgery (DOS). Primary outcome is mobility measured by the Short Physical Performance Battery (SPPB) [13] 4 months after surgery. Secondary outcomes measured at 1, 4 and 12 months postoperatively are place of residence, activities of daily living, balance and gait, falls and fear of falling, quality of life and depressive symptoms, as well as use of health care resources and survival. The complete study protocol has been described previously [14].

During the last years several studies have been performed on treatment of hip-fracture patients by geriatric interdisciplinary teams. The study design including intervention and outcomes have varied and the studies have been performed within different health care systems. To be able to compare studies and evaluate factors of importance for success it is important to describe the interventions in detail. The aim of the present paper is to report the basis for the treatment model in the Trondheim Hip Fracture Trial and to describe treatment options offered to the patients in the DG (experimental group) and the DOS (control group), respectively.

### Basis for the experimental treatment

#### *Model*

During the last years new models in treatment of elderly hip fracture patients including interdisciplinary care and some kind of geriatric intervention have been introduced. The results have been summarized as systematic reviews, guidelines and meta-analyses [15-21].

The models studied have been treatment in orthopaedic wards with geriatric consultant services on request, orthopaedic wards with daily consultative services by geriatricians, initial treatment in an orthopaedic ward with transfer to geriatric wards postoperatively, and treatment in orthopaedic wards where orthopaedic surgeons and geriatricians treat patients together [17,20]. The literature is still inconclusive as to which of these models are most beneficial. However, models with an integrated approach with early involvement of a geriatric interdisciplinary team seem to be superior as compared to models using consultative services or where there is a late involvement of the geriatric interdisciplinary team [17,20].

In the present study the choice of model was based on a review of the literature and also partly being a consequence of a reorganisation in our hospital in 2007. The number of beds was cut down in the DOS reducing the total capacity of the department, therefore temporary solutions were sought to be able to care for the high number of patients admitted with fractures. Therefore, an orthogeriatric bed-unit was established in an acute geriatric ward giving us the opportunity to investigate the potential benefits of performing comprehensive geriatric assessment (CGA) on hip fracture patients in a department previously having shown its efficacy on treating frail geriatric patients in general [9]. The innovative element in this model is a DG being responsible for the medical treatment from admission to discharge, including CGA and initiation of rehabilitation, although most of the rehabilitation program was completed after discharge either at home or in a suitable institution.

To our knowledge this is the first randomised clinical trial of a model treating hip fracture patients pre- and postoperatively performing CGA in a DG with main focus on the intervention during the acute phase.

#### *Comprehensive geriatric assessment (CGA)*

CGA applied on acutely sick, elderly patients treated in specialised geriatric units has been shown to increase the chance of living at home, reduce functional decline and also the risk of nursing home placement [9,10,22,23].

Based upon the evidence from systematic reviews and meta-analyses [22-25] CGA should be performed by an interdisciplinary team of professionals specialised in treatment of elderly patients. Usually the team is comprised of a geriatrician collaborating with nursing staff trained in geriatrics, physiotherapists, and occupational therapists, and in many cases a nutritionist and a social worker. The interdisciplinary team should collaborate both informally and in regular interdisciplinary meetings to discuss the patients, developing individual care plans and defining short- and long term goals for each patient [25].

The assessment should be systematic and multidimensional to identify all relevant problems and initiate adequate assessments. Protocols and assessment tools for common conditions are recommended. Use of care plans is beneficial in order to comply with assessment and treatment. Communication with patients and caregivers throughout the hospital stay is important.

Treatment should be performed in a dedicated unit with sufficient space for patients to move around, offering available aids for mobility and self care, and calendars and clocks as cues for orientation. Discharge planning should start as early as possible. Collaboration across sectors as well as with the patient and her caregivers is necessary to achieve continued rehabilitation and a successful discharge.

In general, CGA should therefore be an optimal tool when treating frail elderly hip fracture patients.

### Developing a treatment program for hip fracture patients in a new clinical pathway

While the DG had extensive experience in performing CGA on acutely admitted geriatric patients, it was not so for the treatment of hip fracture patients within the same context. We therefore had to develop a new program taking into consideration standard routines already established at the hospital on perioperative treatment including anaesthesiological and surgical techniques, and time to surgery. Based on a literature review [16,17,26], the present guidelines of the DOS and a visit to Diakonhjemmet Hospital in Oslo [3] a new program on optimal pre-and postoperative treatment was developed.

We focused at identification and treatment of co-morbidities, pain relief, hydration, oxygenation, nutrition, elimination, prevention and management of delirium, assessment of falls and osteoporosis. Programs for prevention of acute delirium, new fractures, constipation, decubital ulcers, and falls were developed [3,27,28]. A program for early mobilisation and rehabilitation was developed aiming at individualised in-hospital rehabilitation [29,30].

#### *Clinical pathways*

### Patient flow

**Figure 1:**
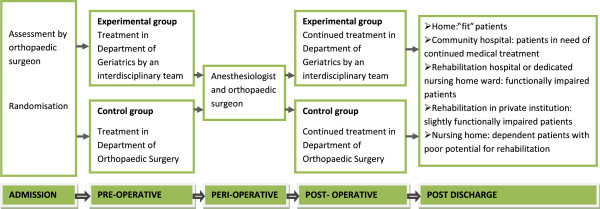
Patient flow in the Trondheim Hip Fracture Trial.

After an orthopaedic resident had diagnosed a hip fracture in the Emergency Room, patients were screened for eligibility in the study. Randomization was performed after the patients had given their informed consent [14]. For patient flow, see Figure 1.

Patients randomized to experimental treatment were transferred to the DG located in the Clinics of Internal Medicine. In Norway geriatric medicine is a branch speciality within internal medicine, and geriatricians and internists were responsible for the treatment in DG. Patients randomized to the control group were transferred to the Trauma Unit located in the DOS, and orthopaedic surgeons were responsible for the treatment.

In both groups patients were transferred to the Operation Theatre for surgery and postoperatively to a recovery unit for observation during the first hours after surgery. Afterwards patients were transferred back to the DOS or DG according to the randomisation.

Patients were discharged from the hospital as soon as they were medically stable after surgery and a suitable place of discharge was available. The DG and DOS dealt with the same municipalities and had the same options for care, treatment and rehabilitation after the hospital stay. After discharge general practitioners and/or doctors at nursing and rehabilitation facilities were responsible for treatment.

Follow-up consultations in the orthopaedic outpatient clinic were decided by the orthopaedic surgeons during the hospital stay and performed in selected patients. There was no follow-up program at the geriatric outpatient clinic. If study patients were referred by general practitioners for assessment in the geriatric outpatient clinic, this was performed “as usual” in both groups. Study-related follow-ups at 1, 4 and 12 months were performed by separate study investigators. A few patients meeting for study related assessments were in need of immediate medical evaluation. These consultations were performed by consultants in the DG.

The major part of the health care system in Norway is organised and financed by the public sector. The government owns and run the hospitals through regional health authorities. General practitioners, physiotherapists and rehabilitation institutions get reimbursement from the government and the regional health authorities. In-hospital treatment and assistance from home care nurses is free of charge. Patients have to cover costs for drugs, physiotherapy and medical treatment up to a total limit of about 200 Euros, above which all is free. Practical help in the patients’ homes are charged according to income. Due to relative low costs for out-patient medical treatment and care the patients’ individual financial situation is normally not determinative for choice of treatment after hospital stay.

### Organization of wards and staffing

The DG consisted of a 10 bed-ward of acute geriatrics services linked to an out-patient facility. During the trial an orthogeriatric 5 bed-unit was established as an additional, but still integrated part of the acute geriatric ward. The DG was located in a new-built part of the hospital. All patients had single-bed rooms. There were no corridor-beds. The department had a separate dining room and the corridors were suitable for moving around. As far as possible an “enriched” environment was created to enlighten the patients’ orientation [28]. This included use of visible calendars and clocks in all rooms, naming plates and signs on the doors, sufficient lightening, and access to necessary aids (including hearing aids) and to news (television, newspapers, and magazines).

In the DOS patients were bedded in the Trauma Unit. During the first part of the study the DOS was located in an old part of the hospital with 19 beds in single-, double- and four-bed rooms with commonly use of additional corridor-beds. The DOS moved into a new hospital building in September 2009 having similar facilities as the DG. At the time of this transfer 219 of 398 study patients had been recruited.

The staff in both departments consisted of doctors, nurses, assistant nurses and physiotherapists. The DG also had occupational therapists. The number of positions per bed for the different professions is shown in Table 1 demonstrating that the DG was generally better staffed than the DOS.

**Table 1:** Organization of treatment in Department of Geriatrics (DG) as compared with the Department of Orthopaedic Surgery (DOS)

	**Experimental group**	**Control group**
**Department**	Clinic of Internal Medicine,	Clinic of Orthopaedics and Rheumatology
	Department of Geriatrics (DG)	Department of Orthopaedic Surgery (DOS)
Facilities^*^	Single bed rooms	Before relocation: single, double or four –bed rooms
		After relocation: single bed rooms
**Number of beds in the ward**	15	19 before / 24 after relocation
**Organization of ward**	5 beds dedicated for hip fracture patients allocated to one single cluster	Hip fracture patients spread among other patients
**Staff working bed-side (number per bed)**	Nurses/assistant nurses: 1.67	Nurses/assistant nurses: 1.48
	Doctors : 0.13	Doctors: 0.11 (0.08 after relocation)
	Physiotherapists: 0.13	Physiotherapists: 0.09 (0.07 after relocation)
	Occupational therapists: 0.13	Occupational therapists: 0

Patients were recruited from April 18^th^ 2008 to December 30^th^ 2010.

^*^DG was located in a new hospital building during the entire study period while DOS was relocated from an old to a new hospital building in September 2009 (as 219 of 398 patients were recruited).

The head of the DOS was involved in planning of the study. Both orthopaedic surgeons and other personnel categories of the DOS participated in training of doctors, nursing staff and physiotherapists in the DG both during the four month run-in period and also the initial part of the study. The educational program involved lectures and bed side practical training. Later the staff in the DG had regular teaching on issues relevant for treatment of elderly hip-fracture patients.

Orthopaedic surgeons assessed patients in DG on request; vice versa geriatricians assessed patients in the DOS on request.

### Standard treatment

In the Emergency Room all patients underwent a standard general clinical examination by an orthopaedic resident, with additional blood samples, measurement of blood pressure, temperature, pulse, oxygenation and an electrocardiogram. Femoral neck fractures were classified according to the Garden classification system [31]. The resident notified the anaesthesiologists and the Operation Theatre.

All patients received intravenous saline or Ringer’s acetate at admission. Low molecular heparin (enoksaparin) was given as thromboembolic prophylaxis from admission to hospital to 14 days after surgery, 20 mg twice daily preoperatively and 40 mg once daily postoperatively . Elastic stockings were used postoperatively if patients had peripheral oedema.

All patients had urinary catheters preoperatively, being removed within 24 hours postoperatively. Pressure relieving mattresses were standard equipment in the new part of the hospital and the nursing staff focused on prevention of decubitus in both groups.

For pre-operative analgesia most patients received femoral nerve blockade. In addition, patients were routinely given paracetamol 1 g every 6 hours during the entire hospital stay, while opioids were given on demand preoperatively and regularly postoperatively.

Preoperatively all patients were assessed by an anaesthesiologist using the American Society of Anaesthesiologists (ASA) score [32]. Minor or moderate medical disturbances did normally not cause delay of surgery, while in cases with medical disorders such as unstable cardiac problems, a severe infection or pulmonary embolism surgery was delayed until stabilisation was achieved. The operability was decided by the anaesthesiologist in collaboration with the orthopaedic surgeon, and in the experimental group also with the geriatrician. Sometimes other specialists were involved such as cardiologists if unstable cardiac disorders or murmurs were found. To avoid complications during anesthesiological and surgical procedures patients using therapeutic doses of heparin, clopidogrel or warfarin were postponed until the risk of bleeding was normalised. Patients using clopidogrel had to wait five days before spinal anaesthesia was considered safe, while patients on warfarin got vitamin K and were ready for surgery when INR was 1.8 or less [33]. Logistic problems within the hospital were the most common cause of delay.

Most patients received spinal anesthesia. For Garden type 1 or 2 fractures most patients were treated with a two-screw fixation but in some few cases hemiprosthesis were used. Garden type 3 or 4 fractures were treated with hemiprosthesis. Pertrochanteric fractures were treated with a sliding hip screw system (Dynamic Hip Compression Screw, DHS, or Compression Hip Screw, CHS). Subtrochanteric fractures were treated either with DHS/CHS (most cases) or antegrade intramedullary nailing. Most patients irrespective of fracture type were allowed full weight bearing postoperatively. Prophylactic antibiotics (cephalotin) were given to all patients, except those getting a two-screw fixation.

Postoperatively the patients were observed in a recovery ward until they were able of moving both legs and their medical condition were stabilised, normally about six hours after surgery.

### Treatment in the experimental group

#### *Comprehensive Geriatric Assessment (CGA)*

CGA was essential in treatment of all patients in the DG (Table 2, Table 3). The aim was to offer as good treatment as possible within available resources, to prevent complications, start rehabilitation as early as possible, and plan for discharge from hospital and further rehabilitation. The work-up focused on assessment and improvement of the patients’ somatic and mental health, functional status and socio-/environmental situation.

**Table 2:** Comprehensive geriatric assessment at the Department of Geriatrics

**Dimensions assessed**	***Somatic health*– concurrent injuries or medical conditions, drug regimen, pain, falls, osteoporosis**
	*Mental health* - cognition, depression, anxiety
	*Function* - ADL, IADL, mobility, sensory loss, elimination
	*Social situation* - place of residence, network, caregiver burden
**Interdisciplinary team work**	Dedicated responsibilities
**Interdisciplinary team meetings**	1^st^ day postoperatively: plan for individual treatment, goal setting, discharge planning,
	4^th^ day postoperatively: evaluation, discharge planning
**Systematic approach**	*Checklists*
	*Treatment protocols*
	*Assessment scales* (Barthel Index, Cumulated Ambulation
	Score: Confusion Assessment Method, Verbal Rating Scale)
**Mobilization/Rehabilitation**	Mobilization out of bed 1^st^ day postoperatively
	Individualised plan for mobilization and participation in ADL being integrated in care plans and ward activities
**Discharge planning**	Collaboration with patient, caregivers and municipality
	Mapping of pre-fracture function, place of residence and social situation
	Discuss discharge destination 1^st^ day postoperatively
	Set realistic short- and long-term goals
	Organize institutional care, aids, assistance, physiotherapy when appropriate

**Table 3:** Medical treatment in the two groups

	** *DOS* **	** *DG* **
Hydration		
Intravenous fluid preoperatively	** *V* **	** *V* **
Monitoring fluid intake postoperatively		** *V* **
Perioperative antibiotic prophylaxis	** *V* **	** *V* **
Thromboembolic prophylaxis	** *V* **	** *V* **
Nutrition		
Assessment of nutritional status*		** *V* **
Nutritional drinks		** *V* **
Decubitus prophylaxis by pressure relieving mattresses	** *V* **	** *V* **
Oxygenation		
Transfusion if Hb < 10		** *V* **
Oxygen if saturation < 95%		** *V* **
Avoiding hypotension (including orthostatic hypotension)		** *V* **
Analgesia		
Femoral nerve block	** *V* **	** *V* **
Paracetamol 1 g every 6 h, opioids on demand	** *V* **	** *V* **
Pain assessment during rest and activity by VRS		** *V* **
Urine		
Removal of catheter within 24 h postoperatively	** *V* **	** *V* **
Screening for infection pre- and postoperatively		** *V* **
Screening for urinary retention		** *V* **
Constipation		
Prophylaxis and monitoring (in cognitively impaired patients)		** *V* **
Delirium		
Regular assessment		** *V* **
Focus on prevention		** *V* **
Osteoporosis assessment		** *V* **
Falls assessment		** *V* **

DOS – Department of Orthopaedic Surgery. DG- Department of Geriatrics

VRS- Verbal Rating Scale.

*Nutritional status – history of recent weight loss, low body mass index, low caloric intake.

The patients’ status before the hip-fracture was mapped by the nurses and the occupational therapists by interview with the patients, and with permission from the patients, also with the care-givers and the Home Services. Information on pre-fracture cognition, activities of daily living (ADL), and instrumental ADL (IADL), mobility, nutrition, living situation, caregiver distress, participation in social activities and assistance needed from the Home services was retrieved. This information was important to make an individual plan for each patient.

The team members in the DG had separate responsibilities. A close formal and informal collaboration between team members was emphasised. Brief interdisciplinary team meetings were held the first postoperative day to discuss the process of early rehabilitation, need of further investigations during the hospital stay, and to set realistic short- and long-term goals and plan for discharge. After three working days a brief follow-up meeting evaluated if progress was as expected aiming at decisions on site of destination and day of discharge.

The in-hospital rehabilitation focused on early mobilization and weight bearing exercise programs, if no restrictions had been made from the orthopaedic surgeon. In addition participation in ADL was emphasized. An individual rehabilitation plan with short term goals was based upon previous function, cognition, type of surgery and motivation. This was integrated with care plans and executed by physiotherapists and nursing staff. Progression was evaluated by the physiotherapists regularly and performance of ADL was evaluated by the occupational therapists on 3^rd^ postoperative day. The long-term goal was to achieve pre-fracture function.

A systematic approach was achieved using check-lists both for each professional category and for the interdisciplinary team work and applying treatment protocols developed for the most common conditions. The following standardised assessment tools were used: Cumulated Ambulation Score (CAS) [34] during the first three days postoperatively. Barthel Index (BI) [35] was scored pre-fracture, and 1^st^ and 3^rd^ postoperative day and at discharge. Verbal Rating Scale (VRS) for assessment of pain [36], Confusion Assessment Method (CAM) was used as screening for delirium [37], Geriatric Depression Scale was used for assessment of depression [38].

Length of stay, discharge destination and necessary arrangements for discharge were discussed within the team and with the patients and their caregivers at several occasions during the stay. Destination for discharge was based upon the patients’ functional and medical status, place of living, and the patients’ and caregivers’ motivation. For patients living in the city of Trondheim (n = 315) a resolution on necessary actions after discharge was made in a discharge planning meeting. Both the patient, his caregivers, representatives for the municipality and nurse and doctor from the DG participated in these meetings. For the other municipalities arrangements were discussed and agreed upon through phone calls with primary health care representatives.

If possible the patients were discharged to their own home with assistance from the Home Services. For these patients physiotherapy was offered to take place either at home, in a physiotherapy clinic or at a day-time rehabilitation centre. Many patients needed institutional rehabilitation and were discharged to private or public inpatient facilities such as rehabilitation wards in hospitals or nursing homes. Some needed continued medical treatment in a community hospital, while some were too sick to be rehabilitated and were discharged to ordinary nursing home wards.

Communication with general practitioners, rehabilitation facilities and nursing homes about individual patients was based mainly upon written discharge reports covering medical treatment, drug regimens, caring needs, physiotherapy and recommended follow-up. At discharge the patients received written reports on their medical situation and drug regimens.

#### *Medical assessment and treatment*

At admission to the DG all patients were clinically examined by a geriatrician or the resident on call and were screened preoperatively by an extensive battery of blood tests, tests of urine and repeated measurements of pulse, temperature, blood pressure and oxygenation. Chest imaging was performed routinely. Medical assessment to reveal concurrent somatic disorders and optimisation of somatic status was emphasised through treatment of medical disorders, electrolyte disturbances, hypoxemia, anaemia and elevated glucose levels.

Hydration and electrolytes were evaluated regularly during the stay. Fluid intake was measured the first days postoperatively, and intravenous fluid was given until patients were able to drink enough.

Oxygenation was measured regularly; oxygen was supplied if saturation was less than 95%. Blood transfusions were given if Hb < 10 g/dl. Monitoring of supine and orthostatic blood pressure was performed. Medication with cardiovascular drugs was adjusted according to these measurements.

To optimise analgesic treatment the nursing staff and physiotherapists assessed pain by using a Verbal Rating Scale (VRS) ranging pain in a scale from one to five during rest and activity [36]. There is no consensus on which opioid should be preferred in frail elderly patients. In DG slow release morphine was the drug of choice; in case of side-effects oxycodone was given instead.

After the urinary catheter was removed on the first postoperative day all patients were scanned with respect to residual urine and checked for infections.

Constipation is very common among hip fracture patients postoperatively. Therefore, preventive treatment with laxatives started the first postoperative day according to a standard procedure. The staff had routines to register defecation of individual patients in order to intensify treatment, especially in patients with cognitive impairment.

Nutrition was in focus both pre-and postoperatively and a specific attention was given to those having a low body mass index, history of recent weight loss or poor appetite. Food intake was monitored if patients were undernourished or had poor appetite. Several patients underwent investigations for pre-fracture weight loss. Many patients were offered specified nutritional drinks until two hours before surgery and protein enriched nutritional drinks and vitamin supplement postoperatively. Meals could be adjusted to each patient’s preferences and needs.

The drug regimen was evaluated in all patients. Preoperatively the following drugs were considered to be withdrawn: antihypertensives, diuretics, all drugs with moderate or strong anticholinergic side-effect [39], and drugs with impact on coagulation. Oral antidiabetic drugs were withdrawn and blood glucose was monitored frequently, insulin was given in reduced doses and on demand. Drugs for heart failure, beta-blockers and antiepileptic drugs were continued, while corticosteroids were given in increased doses if adrenal suppression could be suspected. Postoperatively the entire drug regimen was evaluated with respect to indication, dose, side-effects and interactions.

Confusion Assessment Method (CAM) [37] was used for assessment of delirium. The treatment given to the patients in the DG group has been shown to prevent and/or shorten duration of delirium [28]. Use of aids for impaired hearing and/or vision was used regularly. Repeated information to the patients about their medical situation and encouragement of visits by caregivers was considered important. Oxazepam and/or haloperidol (low doses and short duration) were sometimes used for agitation when pharmacotherapy was considered necessary. If an underlying dementia was suspected the general practitioner was recommended to assess the patient on a later occasion.

Many patients had anxiety and were depressed during the hospital stay. This was mainly treated by caring attempts and occasionally by using oxazepam on demand. If anxiety and depression had been a significant problem before the fracture, medical treatment with SSRI or SNRI was sometimes started.

The falls assessment performed during the hospital stay was based upon the case history from patient and caregivers on previous falls and mobility problems, the cause(s) of the present fall and a clinical assessment with focus on comorbid disorders, drugs, muscle strength and balance according to guidelines developed for the DG.

Many patients already received treatment for osteoporosis. If not, a bone mineral density (BMD) measurement was performed in patients with previous fractures or if the hip fracture was a consequence of a low energy trauma. In case of osteoporosis treatment with calcium and vitamin D was given. Treatment with bisphosphonates (orally or intravenously) was initiated if there were no contraindications and the patient was expected to live long enough to benefit from such treatment.

### Ethics

Participation in the trial was voluntary and according to the Helsinki Declaration. Both oral and written information was given at admission to hospital, later during the hospital stay and at follow up assessments. Written informed consent was achieved from all patients preferably before randomization at admission to hospital, but in a few cases 3 to 5 days afterwards. In patients not being able to write, an oral consent was accepted. Proxies were informed about the study if available, this was especially important if the patient was cognitively impaired. The study has been approved by the Regional Committee of Ethics in Medical Research (Mid- Norway) (REK 4.2008.335). Further details have been described in the protocol article [14].

### Discussion

The present paper describes the rationale behind, and the development and delivery of a new clinical pathway for treatment of elderly hip fracture patients. As far as we know this is the first randomised clinical trial evaluating a treatment model where elderly hip fracture patients are treated in a DG from admission to discharge. The experimental model focused at CGA, fracture specific treatment and initiation of rehabilitation that was continued after discharge from hospital. Statistical analyses on effect of the intervention will be performed later this year.

In our DG we have long-term experience in performing CGA, the efficacy being shown in a study performed 10 years ago [9]. Since then the CGA process in our DG has continuously improved according to recommendations in the Cochrane review by Ellis et al. [25]. The length of stay has been shortened substantially, as well. In theory, the use of CGA should therefore represent an excellent and effective evaluation and treatment option for frail hip fracture patients.

Benefits in favour of DG can only be shown if patient treatment is better than in the DOS. However, treatment of hip fracture patients was introduced into the DG only four months before study start. The competence on medical treatment of hip fracture patients improved rapidly during this piloting and also during the study period. Orthopaedic surgeons were not routinely engaged in the in-hospital follow-up of patients treated in DG which is a potential weak point. Vice versa there has been a quality improvement in treatment of geriatric patients at our hospital during the last 10 years due to extensive involvement of the DG in teaching at hospital level. This is of course of benefit for geriatric patients in general, but brings uncertainties as to the question on whether the new clinical pathway is sufficiently different from “improved” traditional care.

The intention of the present study was to evaluate if the new model would represent a better in-hospital treatment program. Therefore, to avoid a mix of in-hospital and post-discharge interventions the trial focused on CGA and rehabilitation during the hospital stay with no specific follow-up after discharge. Potential effects of the in-hospital CGA on the primary endpoint of mobility may therefore be lost when measuring for potential benefits 1, 4 and 12 months after discharge.

The Cochrane review by Ellis and co-workers did not show targeting to be essential for outcomes of CGA [25]. Our study population is a case-mix of both healthy and frail participants, although nursing home patients and patients being unable to walk 10 m were excluded due to choice of mobility and place of residence as endpoints. Intuitively we still think that frail hip fracture patients would potentially benefit more from CGA than the non-frail, and that CGA would potentially be beneficial also for patients excluded from the study.

Both the DG and the DOS are treating acutely sick patients and therefore considerably better staffed than for example the ward in Stenvall’s study [27]. In our hospital the nursing staff also has to take responsibility for kitchen work and household (except cleaning of floors), thus requiring more nursing staff. The present paper shows that the DG is somewhat better staffed than the DOS. This may of course be explained by an ambitious CGA program. However, other aspects may also have impact i.e. the case-mix in the DG is in general more frail and complex, with almost all patients needing help in ADL, and most of them are cognitively impaired. The DG staff is also extensively involved in supervision and teaching obligations outside the ward and the hospital.

The last follow up sessions in the study are recently finished and the results will reveal if the experimental clinical pathway will be beneficial for both hip fracture patients and society.

### Availability and requirements

Project name: the Trondheim Hip Fracture Trial (Clinical Trials Gov NCT00667914)

Project home page: none

Operating system: n.a.

Programming language: n.a.

Other requirements: n.a.

License: n.a.

Any restrictions to use by non-academics: None

## Abbreviations

ADL, Activities of Daily Living; ASA, American Society of Anaesthesiologists; CAM, Confusion Assessment Method; CAS, Cumulated Ambulation Score; CGA, Comprehensive Geriatric Assessment; CHS, Compression Hip Screw; DG, Department of Geriatrics; DHS, Dynamic Hip Compression Screw; DOS, Department of Orthopaedic Surgery; IADL, Instrumental Activities of Daily Living; SPPB, Short Physical Performance Battery; VRS, Verbal Rating Scale.

## Competing interests

The authors declare that they have no competing interests.

## Authors’ contributions

IS participated in planning research design and performance of the study, was responsible for making clinical guidelines and is the primary author of this paper. OS initiated the study and has been the project leader; he also led the work on implementing the intervention in the Department of Geriatrics (DG). AP has been participated in planning the study, treating patients in DG, completing data files and will be responsible for analysing the data. EE has been participating in planning and performing the intervention at the DG. JLH has made substantial contribution to plan and perform the study including research design, intervention, completing the data base. LGJ has contributed in teaching and supervising the staff at DG with fracture-related treatment. All authors have contributed to the drafting of this paper and read and approved the final version.
